# An Extended Approach to Predict Retinopathy in Diabetic Patients Using the Genetic Algorithm and Fuzzy C-Means

**DOI:** 10.1155/2021/5597222

**Published:** 2021-06-26

**Authors:** Saeid Jafarzadeh Ghoushchi, Ramin Ranjbarzadeh, Amir Hussein Dadkhah, Yaghoub Pourasad, Malika Bendechache

**Affiliations:** ^1^Faculty of Industrial Engineering, Urmia University of Technology, Urmia, Iran; ^2^Department of Telecommunications Engineering, Faculty of Engineering, University of Guilan, Rasht, Iran; ^3^Department of Electrical Engineering, Urmia University of Technology, Urmia, Iran; ^4^School of Computing, Faculty of Engineering and Computing, Dublin City University, Ireland

## Abstract

The present study is developed a new approach using a computer diagnostic method to diagnosing diabetic diseases with the use of fluorescein images. In doing so, this study presented the growth region algorithm for the aim of diagnosing diabetes, considering the angiography images of the patients' eyes. In addition, this study integrated two methods, including fuzzy C-means (FCM) and genetic algorithm (GA) to predict the retinopathy in diabetic patients from angiography images. The developed algorithm was applied to a total of 224 images of patients' retinopathy eyes. As clearly confirmed by the obtained results, the GA-FCM method outperformed the hand method regarding the selection of initial points. The proposed method showed 0.78 sensitivity. The comparison of the fuzzy fitness function in GA with other techniques revealed that the approach introduced in this study is more applicable to the Jaccard index since it could offer the lowest Jaccard distance and, at the same time, the highest Jaccard values. The results of the analysis demonstrated that the proposed method was efficient and effective to predict the retinopathy in diabetic patients from angiography images.

## 1. Introduction

Patients suffering from diabetes, in some cases, lose their eyesight, which is often because of diabetic retinopathy (DR), though, there are some other causes of vision loss or poor eyesight, including other retinal and nonretina vision conditions such as macular degeneration and glaucoma (which are associated with age) and neuropathy vascular vision (which is Nonarteritic Anterior Ischemic Optic Neuropathy (NAION)) and cataract [1–4]. In addition, when an individual (a patient) with diabetes complains of visual disturbance, despite the 6.6's precision, the refractive errors, contrast sensitivity, direct light, and compliance range need to be taken into account. The doctors managing diabetic patients should consider these vision problems in order to make sure that timely referral and treatments are done to control vision impairment as much as possible. This control significantly affects peoples' daily life, particularly for activities like driving [5, 6].

Diabetic retinopathy can be considered a disease of the type of diabetes mellitus (DM), and it has been recognized as the primary basis for blindness in healthy adults in advanced countries. The retinal and choroidal circulation in the eye changes with the diabetic retinopathy. Multiple vascular dysfunctions may be the primary DR markers [7–9]. Also, Nonproliferative Diabetic Retinopathy (NPDR) can be defined by loss of capillary protein, thickening morphology of the glomerular basement membrane, and loss of smooth muscle cells, which leads to micro aneurysm, which is an essential indicator of the prediction of DR's progress [10, 11].

Additionally, in the foveal avascular zone (FAZ) and capillary outflow in florfenicol angiography (FA) studies, patients with primary DR have been observed. Recently, OCT angiography has shown the presence of microenvironment and capillary excision in patients with diabetic retinopathy. Although DR is generally known as a retina, histology, electron microscopy, and green and endogenous angiography (ICGA), studies have shown that diabetes with degeneration of cholangiocarcinoma (CC) and also the formation of laminar deposition the term “diabetic cryotherapy “ is used to refer to changes in the CC associated with diabetes [5, 12, 13]. Imaging of retinal and choroidal ducts in diabetic patients does not occur regularly, especially in patients referring to primary care centers, since color-based angiographic techniques, such as FA and ICGA, require an intravenous injection of external dyes [14].

In addition, considering its location and structure, CC visualization is challenging with both FA and ICGA. OCTA is a relatively new imaging technique that allows for indirect visualization of the retina and CC pathways. CATA is based on the contrast of the motor and, contrary to the FA and the ICGA, does not need to inject different colors [14, 15]. The OCTA B-scan is produced by acquiring OCT B-scans repeated in a fast series of a retina. If the texture is constant, repeat scans B are identical; however, if there is a flow of blood, moving erythrocytes create pixels in OCT B-scans that are converted to the amount of current flow rate. The volume of OCTA can be generated by acquiring redundant B-scans in several retina networks. Since the depth of the OCT depth has been resolved, the various vascular layers of the retina can be individually visualized, which is not possible with color-based angiography [2, 4, 16]. In addition, since it is undeniable, the OCTA can be repeated several times in subsequent reviews or even during the same visit. Finally, since OCTA is the standard OCT imaging format, structural information is obtained at the same time and is inherently recorded by angiography information. This feature provides a simultaneous representation of both structural and angiographic data [2, 4, 16].

Diabetes types I and II increase more not only in the elderly but also in the people suffering from obesity and also the young adolescents. Diabetes is a disease of multiple systems; thus, each part of the eye can be vulnerable. Although eye complications have been reported frequently by doctors for over two centuries, DR has played a significant role in its destructive impacts that result in the early loss of sight [15, 16]. DR is common today in the world, and prevention is difficult for people. The ophthalmologists typically detect the DR severity degree by various visual tests of the fundus through directly examining and evaluating the color photographs. A challenge in this sense is that this process is both time-consuming and costly. The diagnosis of DR and diagnosis of primary diseases remain somewhat personal, with statistics of agreement between trained specialists in studies previously recorded being different [2].

Additionally, 75% of patients with DR condition are currently living in underdeveloped or poor regions, where adequate doctors, medical health care facilities, and diagnostic infrastructures are not available. To control the rate of the increasing number of the DR patients which can lead to blindness all over the globe, many global screening solutions have been set up to deal with the spread of disease inside the eye and better maintain vision; however, such programs make extensive use of DR to diagnose and treat retinopathy and related problems on an individual basis in an efficient way [4, 15]. As a result, millions across the world are currently exposed to visual impairment without recognizing adequate eye care. For the aim of overcoming the current problematic conditions, automated solutions have been proposed for the diagnosis of retinal disease using a dyed fundus illustration. In fact, those methods that are based on only some obtained samples from different patients in different times in one medical clinic or screening center are not applicable to fundus images [2, 16]. This is due to the fact that various kinds of fundus cameras are used in different clinics for detecting eye dilatation. In addition, many of these algorithms are employed to manually extract the attributes related to the DR using some hand-crafted features, aimed at describing the prediction of anatomical structures in the fundus, such as optical discs, blood vessels, or macula. Though these hand-crafted representations might be run on the Foundation Individual Dataset, they are once again trying to accurately detect DR through fundus images that are tailor-made for different demographic purposes [15, 17]. The general-purpose features, comprising GLCM (Gray Level Cooccurrence Matrix) [18], GLRM (Gray Level Run Length Matrix) [19], and Histogram of Oriented Gradients (HOG) Histogram of Oriented Gradients, have been examined using nonspecific methods applicable to specifying DR properties; however, they have shown weaker and disproportionate properties, which cannot describe the nuances of retinopathy.

Regular screening of diabetic patients for DR is a highly technical and critical aspect of patient care. The care and timing of this care are very important for the cost of the treatment. If timely diagnosed, DR compensatory treatments are reachable, and this is a pivotal process for anyone who needs them. The DR classification involves weighting many features and the position of these features. This process is an arduous task for doctors and needs a lot of time. After training, systems can acquire much more rapid classifications, and this will enable them to help clinicians in a quick classification [17, 20, 21].

DR can be described by morphological lesions associated with abnormalities in the retinal blood flow. These lesions represent a regional distribution that encompasses risk factors in the initial phases of the disease and can predict disease progression. Moreover, this disease encounters two different threatening symptoms of Diabetic Maculopathy (DM) vision and Proliferative Diabetic Retinopathy (PDR) in the late stages of the disease. These symptoms can be identified through considering the difference in spreading and category of lesions [4, 20]. The progression of diabetic retinopathy is also associated with sectional changes in the retinal blood flow and the adjustment of the diameters of the retinal capillaries in the macular region and in the retinal environment. The literature consists of some research into the segmentation of images in medical research such as the fuzzy clustering method with residual driving and automatic fuzzy clustering methods [22, 23]. Algorithms that are without supervision do not involve training networks; they are called clustering methods, e.g., Fast FCM, and Robust FCM [24–29].

On the other hand, methods with supervision (e.g., ANN and recurrent convolution neural network) train an optimum network for detection and segmentation of medical images [30–34]. Unsupervised methods usually are faster than training methods and are typically used as a preprocess for supervised methods. Methods such as FCM [21, 35], KNN [21, 36], and SVM [37] are commonly used as automated methods for generating ground truth data for automated methods. The advantage of automated methods like FCM is the detection of locations with distinguished color [29, 38]. In other words, tumors should be light or dark and different from other pixels. Gadekall et al. [39] suggested a deep neural network model based on a Grey Wolf Optimization (GWO) technique to classify the extracted features of diabetic retinopathy dataset. Employing the GWO can be beneficial to find optimal parameters for training the DNN structure. Behera and Chakravarty [40] utilized a scale-invariant feature transform (SIFT) approach to extract some key features. Also, to capture the exudate regions on each retinal images, Speeded Up Robust Features (SURF) has been employed.

In this study, the growth region technique based on the combination of the FCM and Genetic Optimizer Algorithm (GA) strategies for the aim of diagnosing diabetes is proposed, considering the angiography images of the patients' eyes. The main novelty of FCM and GA is the optimization of automated methods of clustering images. Several previous works are providing automated methods for the extraction of ground truth images. However, some are not flexible and need further improvement. The methods presented in this paper without using hand methods exploited a genetic algorithm to find the best initial seeds of FCM method in the growth region algorithm.

## 2. Extended Growth Region Method

### 2.1. Data Collection

**Figure 1:**
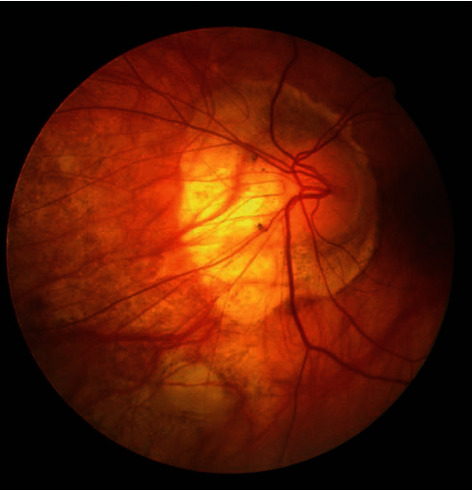
Example of angiography image.

The present paper was designed to assess the dimensions and shape of FAZ in patients with diabetic retinopathy in comparison with healthy controls, using OCT angiography. The database contains 224 images of a fluorescence-sized angiography, 250 × 250 depths of 8 bits per pixel, and each pixel size of 11 × 11 micrometers taken from the eye of 14 times over a different period. Among them, 12 are diabetic, and two are nondiabetic, with an average of 16 images per person (192 images from diabetic and 32 images from nondiabetic eyes). Indeed, 12 of the images are obtained from the right eye and 2 from the left eye. The Heidelberg Specialist apparatus performed imaging in fluorescing angiography from Urmia Hospital. These images are in the jpeg format.  Figure 1 displays an instance of a database. The Heidelberg device is a prototype device with a bandwidth of 50 nm, a lateral resolution of 14 *μ*m, and an axial resolution of 7 *μ*m that obtain 85,000 A-scans per second. This device produced by Heidelberg Engineering (Heidelberg, Germany) and employs an amplitude decorrelation technique for applying to a volume scan on a 15 × 10° or 15 × 5° zone including an area with the size of 4.3 × 2.9 mm or 4.3 × 1.5 mm.

### 2.2. Image Processing

In recent years, image processing has been extensively used, especially with the advent of advanced techniques such as discriminatory information processing, e.g., digital cameras and scanners. On the other hand, the images resulted from these techniques are generally associated with different degrees of noise, and even sometimes, these techniques fail to fade boundary inside an image [33, 41–43]. This problem finally decreases the resultant image resolution. In this context, image processing refers to all operations and techniques adopted by users for the purpose of decreasing the defects and increasing the image quality.

The region's growth method is an attempt for splitting the image into discrete regions on the basis of the degree of homogeneity or similarity between two or more parts of the image in neighboring pixels; thus, at another level, it depends on the criteria applied to homogeneity analysis [44, 45]. The pixels in each area are gathered together using some specific criteria: illumination, color, etc. This growing-base strategy is a simple method in the category of area-based methods and is based on the testing the intensity of an initial pixel with all touching pixels and adds them to the first pixel to search again for finding other pixels that belong to the segment. In the image segmentation context, the histogram-based methods are only focused upon the distribution of image pixels in the gray level, whereas the local growth strategies consider that the neighboring pixels also possess adjacent gray levels [44, 46].

In the following, the way the area-based methods work is explained step by step [47, 48]:

1. The number of initial seeds is the beginning of the algorithm
2. With the use of these seeds, the regions start their growth, and the pixels that resemble the original pixels will be added to that area
3. Once the growth of area stops, the subsequent grain is taken into consideration, and the next area growth continues
4. The above-mentioned steps will be continued until all of the pixels that exist in the image belong to one area

**Figure 2:**
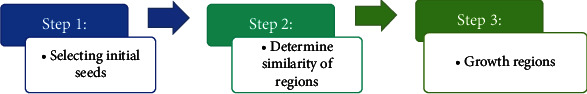
The region growth method steps.

The area's growth method has the following steps (Figure 2).

### 2.3. Selecting Initial Seeds

For the algorithm to get started, the initial seeds need to be manually entered. In this state, the algorithm begins operating by choosing the user's initial points. Several methods in the field have the capacity of taking the start locations without having any prior knowledge about it. For instance, a random step strategy can be employed to explore the first points [47, 49].

For the aim of choosing the initial points, the present study proposes a new model using the combination of fuzzy clustering (FCM) and genetic optimization (GA) approaches. As in a FCM method every pixel inside the image can belong to more than a cluster, it helps to detect the border of any objects more accurately. Initially, a clustering process is carried out on the input image by applying the fuzzy clustering technique. This clustering method can be implemented based on the defining the membership grade *F*_*M*_ and cluster centers *F*_*C*_. So, these parameters can be selected based on a trial and error method or by and optimization approach [29, 50]. To address the problem of experimenting all possible values for the parameters to obtain the maximum segmentation accuracy, the GA responsible for minimizing the cost function (sum of squares of the error) and determining the best value for these parameters. The cost function *E* is demonstrated in Equation (1) [51, 52]:

(1) $$\begin{array}{c} E\left(C\right)=\overset{n}{\underset{j=1}{\sum }}\overset{K}{\underset{i=1}{\sum }}m_{ij}{\left\|x_{j}-c_{i}\right\|}_{A}^{2}, \end{array}$$

where the relation *m* can be expressed as follows:

(2) $$\begin{array}{c} 0<\overset{n}{\underset{j=1}{\sum }}m_{ij}<n,\;j=1,2,\cdots ,K,\\ \overset{k}{\underset{i=1}{\sum }}m_{ij}<1,\;j=1,2,\cdots ,n,\\ \overset{k}{\underset{i=1}{\sum }}\overset{n}{\underset{j=1}{\sum }}m_{ij}=n. \end{array}$$

Moreover, *m* and *C* are computed employing Equation (3), as follows:

(3) $$\begin{array}{c} m_{ij}={\left[\overset{K}{\underset{k=1}{\sum }}\left(\frac{{\left\|x_{j}-c_{i}\right\|}_{A}}{{\left\|x_{k}-c_{i}\right\|}_{A}}\right)\right]}^{-1},\;1\leq i\leq K,1\leq j\leq n, \end{array}$$

(4) $$\begin{array}{c} c_{i}=\frac{\sum_{j=1}^{n}m_{ij}x_{j}}{\sum_{j=1}^{n}m_{ij}},\;1\leq i\leq K. \end{array}$$

### 2.4. Determine the Similarity of Regions

After the determination of the initial points during the above step, the criterion of similarity is chosen for the regions. The objective of using this criterion is to examine all pixels around the new attached pixels to decide whether they can be added to the working area or not. This procedure specifies the attribution of the novel pixel to the corresponding area [53, 54].

A similarity criterion, which is widely used in this field, is the standard deviation that can be applied to each evaluating segment, meaning the new pixel *I*_*n*+1_(*x*, *y*) needs to be added in the segment area if it can pass the following condition:

(5) $$\begin{array}{c} \mu_{n}-X\sigma_{n}<I_{n+1}\left(x+y\right)<\mu_{n}+X\sigma_{n}, \end{array}$$

where *σ*_*n*_ stands for the standard deviation, *μ*_*n*_ demonstrates the mean, and *X* illustrates a weighting parameter used for defining how many pixels are different within the region. In general, in case *X* = 3, more likely all pixels (about 99.70 percent) of the evaluating locations (pixels) are chosen in the same segmented area. It should be mentioned that whatever the value of *X* is smaller, the chance of finding more similar pixels in a vicinity is lower. In another word, by defining the low and high values for the parameter *X*, the number of the segmented area inside the image will be higher and lower, respectively. In addition, another criterion that needs to be considered is the image mean level (256 possible intensity levels). For doing this, the mean of the area which has been segmented by this technique is calculated, and then the intensity value of the new evaluating pixel *I*_*n*+1_(*x*, *y*) for assessing based on the Equation (6) is extracted. This means that Equation (6) must be true for adding the new pixel in the segmented area before.

(6) $$\begin{array}{c} \mu_{n}-X<I_{n+1}\left(x+y\right)<\mu_{n}+X. \end{array}$$

### 2.5. Growth Region

When the initial seeds are determined for the algorithm commencement and also the similarity criterion is determined for pixels with areas, then, the area growth process gets started. The area growth, which starts from the initial seeds, is done through choosing the neighboring regions [53, 54].

#### 2.5.1. Fuzzy C-Means (FCM)

Grouping similar data or pixels in the same group (segmented data or pixels) using machine learning techniques based on some criteria can be defined as clustering [38, 55–59]. This diving data can be implemented by many approaches such as DBSCAN, fuzzy C-means, mean shift, and K-means algorithms. In the fuzzy C-means and K-means approaches, first, a number of groups/classes need to be defined, but it is not true when working with the mean shift and DBSCAN techniques (algorithms calculate the optimum number of the clusters) [43, 60].

The FCM method is based on finding the similarity between data points inside the image to define the groups using an unsupervised manner. Unlike some approaches such as K-means and C-means, in this method, each point is belonging to some clusters based on a weighting parameter. The objective function that needs to be minimized using FCM strategy is demonstrated in Equation (7) [38]:

(7) $$\begin{array}{c} E=\overset{m}{\underset{k=1}{\sum }}\overset{N}{\underset{i=1}{\sum }}{\text{mem}}_{ki}{\left\|{\text{center}}_{k}-\text{pixe}{\text{l}}_{i}\right\|}^{2}, \end{array}$$

(8) $$\begin{array}{c} {\text{Mem}}_{ki}=\frac{1}{{\left(\sum_{j=1}^{m}\left({\text{center}}_{k}-{\text{pixel}}_{i}\right)/\left({\text{center}}_{j}-\text{pixe}{\text{l}}_{i}\right)\right)}^{t}}, \end{array}$$

(9) $$\begin{array}{c} \overset{m}{\underset{k=1}{\sum }}{\text{mem}}_{ki}=1,\;{\text{mem}}_{ki}\epsilon \left[0,1\right], \end{array}$$

where pixel_*i*_ stands for the *i*th sample of *I*, center_*k*_ refers to the centre of the *k*th cluster, mm_*ki*_ stands for the membership score for the *i*th sample that relates to the *k*th divided group of pixels (clusters), *m* depicts the number of clusters, and *N* is a number for demonstrating how many pixels present in image *I*.

#### 2.5.2. Genetic Algorithm (GA)

Object detection or object recognition can be conducted by extracting key features of the image related to each possible object in the image. It means each pixel inside the image based on adjacent pixels can extract a lot of local or global features even by changing the image representation such as Local Directional Pattern (LDP) [61, 62] or Local Binary Pattern (LBP) [41, 63]. So, by investigating these features, we can find the structure and shape of each object in the image. This investigating process can be conducted by an optimization process to decrease the time of the consideration [64–67]. The genetic optimization algorithm was designed on the basis of the evolution theory and the survival of the fittest or natural selection proposed by Charles Darwin. This popular algorithm has been extensively applied to optimization problems [64]. In the genetic algorithm, at each implementation stage, random processing is performed on a group of search spots. It means a lot of random initial points are evaluated for finding the best possible points to apply in the next step. This way, each point is assigned with a sequence of traits, and then, the sequences are exposed to genetic operators. Next, based on minimizing the cost function (or increasing the fitness function), these obtained sequences are divided to separate parts and then each part adds to another part to create a new chain of spots within the searching space until stopping criteria are reached. The task of dividing and then adding sequences is done by considering their participation probability [68–70]. In the present research, the cost function is computed based on the difference between the input image and the image attained by the region growing technique. It gets started from the initial random point, as can be expressed in the following relation:

(10) $$\begin{array}{c} \cos t={\left(\text{GM}-I_{1}\right)}^{2}+{\left(\text{WM}-I_{2}\right)}^{2}+{\left(\text{BM}-I_{3}\right)}^{2}, \end{array}$$

where WM, BM, and GM demonstrate the white matter, black matter, and gray matter, respectively, and *I*_1_, *I*_2_, and *I*_3_ represent the images acquired from segmentation process.

#### 2.5.3. Performance Analysis

The Specificity, Precision, Accuracy, False positive rate (FPR), Sensitivity, Relative volume difference (RVD), Volume overlap error (VOE), and Dice similarity (DICE) are the eight evaluation metrics that were employed to assess the result of the proposed model (can be calculated using Equations (11)–(18)). Sensitivity or true positive rate refers to the percentage of the important objects that identified correctly. Specificity refers to the percentage of the unimportant objects (healthy tissue) identified as unimportant correctly. Accuracy (measure of statistical bias) and precision (measure of statistical variability) represent the closeness of the measurements to a predefined value and to each other, respectively [38, 41, 71, 72].

- True Positive (TP): the important object (retinopathy) is recognized perfectly.
- True Negative (TN): the healthy tissue is recognized perfectly.
- False Positive (FP): the important object is recognized with mistakes.
- False Negative (FN): the healthy tissue is recognized with mistakes.

(11) $$\begin{array}{c} \text{TPR or Sensitivity}=\frac{\text{TP}}{\text{FN}+\text{TP}}, \end{array}$$

(12) $$\begin{array}{c} \text{TNR or Specificity}=\frac{\text{TN}}{\text{FP}+\text{TN}}, \end{array}$$

(13) $$\begin{array}{c} \text{PPV or Precision}=\frac{\text{TP}}{\text{FP}+\text{TP}}, \end{array}$$

(14) $$\begin{array}{c} \text{FPR or False positive rate}=\frac{\text{FP}}{\text{FP}+\text{FN}}, \end{array}$$

(15) $$\begin{array}{c} \text{ACC or Accuracy}=\frac{\text{TP}+\text{TN}}{\text{All results}}, \end{array}$$

(16) $$\begin{array}{c} \text{Dice}\left({\text{set}}_{1},{\text{set}}_{2}\right)=100\times \left(\frac{2\times \;|\;{\text{set}}_{1}\cap {\text{set}}_{2}\;|\;}{\left|{\text{set}}_{1}+{\text{set}}_{2}\right|}\right), \end{array}$$

(17) $$\begin{array}{c} \text{VOE}\left({\text{set}}_{1},{\text{set}}_{2}\right)=100\times \left(1-\frac{{\text{set}}_{1}\cap {\text{set}}_{2}}{{\text{set}}_{1}\cup {\text{set}}_{2}}\right), \end{array}$$

(18) $$\begin{array}{c} \text{RVD}\left({\text{set}}_{1},{\text{set}}_{2}\right)=100\times \left(\frac{{\text{set}}_{1}-{\text{set}}_{2}}{{\text{set}}_{2}}\right), \end{array}$$

where set_1_ and set_2_ represent the segmentation and ground-truth results, respectively.

In a given test, specificity and sensitivity are dependent upon the nature of the test and the type of test used. This is worth mentioning that a test result is not interpretable using Specificity and Sensitivity only. Receiver Operating Characteristic curve (ROC curve) is a probability curve that refers to a graphical plot (TPR or true positive rate against the FPR or False positive rate; FPR is on the *x*-axis and TPR is on *y*-axis) for checking any classification model's performance at various thresholds settings.

## 3. Experimental Results and Discussions

As the region growing algorithm is based on finding the homogeneity defined by calculating the pixel intensity statistics, the first step in the region growing is to identify a set of seed positions (initial set of small areas). The initial region starts as the exact location of these seeds that are based on some user criterion or can be selected based on the generating random number. The regions are then grown from these seed points to other points in the vicinity of pixels depending on a region membership criterion (pixel intensity). To determinate whether the new point is good enough to join the selected seed location (point) or not, the mean and standard deviation of the growth region need to be computed, as depicted in Equations (16) and (17).

Here, we use 8-connected neighborhood for our pixel's adjacent relationship to grow from the seed points. By examining the value of the pixels in the vicinity of the seed locations, all tested pixels are categorized into (1) seed points and (2) background. It is an iterated process until there are no changes in two successive iterative stages. The number and location of these initial location are determined by GA. By examining the random values for both the location and number of the seed points obtained by GA, the algorithm calculates cost function for each of them. Then, those locations that reach the best result can be selected for the final segmentation using region growing approach. This process is expressed in the following relations:

(19) $$\begin{array}{c} \mu_{N}=\frac{\left(N-1\right)\left(\mu_{N-1}\right)+I_{N}}{N}, \end{array}$$

(20) $$\begin{array}{c} \sigma_{N}=\sqrt{\frac{\left(N-2\right){\left(\sigma_{N-1}\right)}^{2}+\left(N/N-1\right){\left(I_{N}-\mu_{N}\right)}^{2}}{N-1}}. \end{array}$$

Afterwards, for the neighboring points that have been inserted to this class, they will also be found in the same neighborhood, and then the update of the parameters is done. The searching process is continued until the detection of the first class is completely done, and no other pixel is appended.

**Figure 3:**
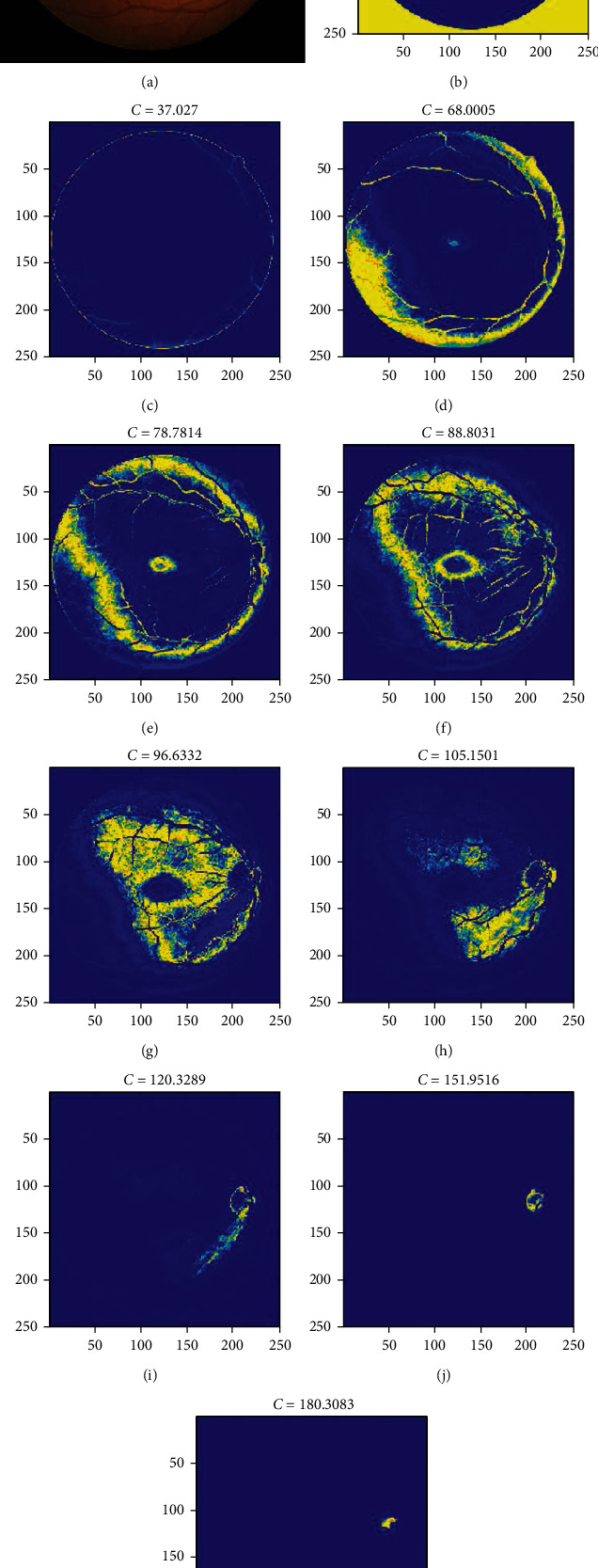
The results of the presented method for detection of retinopathy.

**Figure 4:**
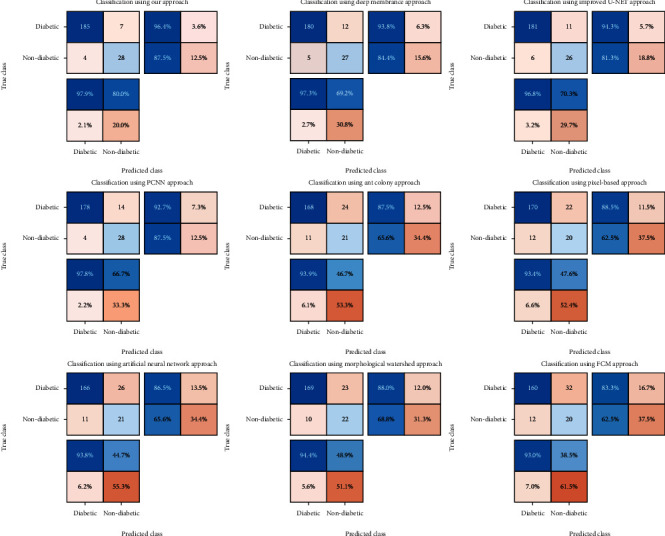
Demonstration of confusion matrix for our approach and 8 other methods.

The numbers of clusters in FCM are 6-10, and for the genetic algorithm, the population is fixed at 20, and the mutation rate is set to 0.2.  Figure 3 demonstrates the results of the clustering process performed with the use of the proposed FCM-GA method. As clearly shown, the yellow areas (some similar parts of the image) are decreasing inside the loop until reaching the best possible solution. Regarding Figure 3(a), the retina target region is almost above the right part of the image with high contrast. After classification using the FCM method, the results of the membership function are depicted as can be seen in Figure 3. The final image could detect the precise situation of the retina target region. The confusion matrix for testing the dataset is demonstrated in Figure 4.

The TPR (Sensitivity), ACC (accuracy), TNR (Specificity), PPV (Precision), and FPR (False positive rate) values using some approaches are described in Table 1. As it is clearly shown, our approach gains the best scores among all evaluated methods. The Deep membrane and Improved U-NET obtain the significant value in ACC criteria. The worst result (except FPR) was reached by the Artificial Neural Network (ACC = 83.4, TPR = 93.8, TNR = 44.7, PPV = 86.5). Besides, the PPV values of the Improved U-NET and Deep membrane are considerably higher as compared to the Ant colony, Pixel-based Segmentation, Morphological Watershed, Artificial Neural Network, and FCM results. For the TPR values, there is a small difference between Deep membrane and PCNN models. The FPR and TPR values of the Deep membrane model is partly similar to the proposed algorithm; however, Deep membrane approach has a meaning unlike TNR. Also, the Pixel-based Segmentation method represents the worst result in terms of the FPR. The Ant colony, Pixel-based Segmentation, Morphological Watershed, Artificial Neural Network, and FCM methods obtain the worst results in term of TNR, whilst their TPR values are acceptable.

Table 2 illustrates the evaluation of our segmentation technique and the results from recently published models. The attained values of RVD measurement imply the amount of oversegmentation or undersegmentation. Therefore, a zero score stands for the best possible segmentation result, whilst a negative value represents the segmented result image is not as large as that of the corresponding actual annotated image. DICE equals one for a precise segmentation. The Dice scores of the Deep membrane and PCNN models are the same and are partly similar to those of the proposed algorithm; however, these two techniques have a significantly different RVD. Also, the Deep membrane and Pixel-based Segmentation algorithms show the undersegmentation results with -2.46 and -5.64 values, respectively. Besides, the RVD values of the Artificial Neural Network and Morphological Watershed models were considerably higher as compared to our outcomes. Our strategy and Ant colony algorithm have significantly different RVDs [19]. Considering the VOE, the lowest and highest values belong to our model and Artificial Neural Network, respectively. Moreover, there are minimum differences between VOEs in Ant colony, Pixel-based Segmentation, and Artificial Neural Network techniques.

Therefore, the division of an ideal abnormal area into angiography images plays a vital role in medical imaging programs. Segmentation algorithms are applied to promoting the precision and function in case of images in medical contexts. The method proposed in this paper was applied to a number of images, and also, the actual image analyses were compared to each other in order to examine the results of the reading. This study conducted comparative research on various performance criteria. Generally, in the GA classification, FCM returned more accurate results. Extending this work to improve GA segmentation with some other algorithms, without changing the primary nature of medical images, is required.

As the obtained results indicate obviously, the proposed method performed successfully in reducing the VOE score. It indicates the fact that the image has been split with higher precision. In the present research, the initial points were segregated automatically with the use of the region growth method. In addition, a genetic algorithm was employed for the purpose of selecting the most proper initial points in an automatic way. The GA approach was utilized, and the best possible fitness function was defined for the aim of image segmentation. This way, the proper initial locations were determined to get the algorithm started. At the final step, the images were exposed to the proposed algorithm, and the obtained outcomes were compared to those of the growth method of the area wherein the manually chosen points were chosen. Findings confirmed the capability of our technique in decreasing the fragmentation errors.

In the present study, a total of 224 images of the retina were employed, which included 192 images from diabetic and 32 images from nondiabetic eyes. We used 70%, 20%, and 10% of total data for training, validation, and test, respectively. The proposed method showed the highest level of sensitivity among all. That indicates that GA can be used more effectively in selecting initial points.

## 4. Conclusions

In the present paper, a segmentation approach using the growth region technique is represented. This model is based on the combination of the FCM and GA methods for the purpose of diagnosing diabetes from the angiography images of the patients' eyes. The algorithm started with the early locations inside the image, and the mean of these locations were assumed as the mean of the objective area, and the early value for standard deviation was assumed as zero. For detecting the extract target area, the retinopathy images were recommended with the employed dynamic image analysis on the basis of the genetic algorithm. The eight evaluation metrics including Specificity, Precision, Accuracy, False positive rate (FPR), Sensitivity, Relative volume difference (RVD), Volume overlap error (VOE), and Dice similarity (DICE) were employed to assess the result of the proposed structure. The fuzzy fit function in GA was compared to another technique, and the outcomes indicated that the proposed method was more suitable to the sensitivity and Dice measures, with the maximum sensitivity and dice index. Considering the VOE index, the lowest and highest values are related to the suggested model and Artificial Neural Network, respectively. Additionally, there are minimum differences between VOEs in the Artificial Neural Network, Pixel-based Segmentation, and Ant colony techniques.

The fuzzy fitness function c-means evaluate the segmentation of threshold-based physical fuzzy tools, the fuzzy C-means tool in GA. The method proposed in the GA classification returned more proper results in its general performance, extending this to improve GA segmentation with some other algorithms, without changing the nature of the main medical angiography images is required. As the results demonstrated, the method introduced in this study was successful in decreasing the value of the VOE and RVD indexes. It indicates that the image has been split with higher precision.

Furthermore, a genetic optimizer was employed in this study in order to apply the proper early locations for the segmentation task automatically. The suitable early points were identified to start the algorithm with the use of GA and through giving a definition for the proper fitness function for the image segmentation purposes. At the final step, the prepared images were exposed to the proposed algorithm, and the obtained outcomes were compared with the growth manner of the region where the points are chosen manually. The obtained results confirmed the high capacity of our proposed algorithm in terms of reducing fragmentation errors.

## Figures and Tables

**Table 1:** Comparative outcomes of the proposed strategy and recently published studies.

Method	ACC	TPR	TNR	PPV	FPR
Deep membrane [4]	*92.4%*	97.3%	69.2%	93.8%	84.4%
Improved U-NET [6]	*92.4%*	96.8%	70.3%	94.3%	81.3%
PCNN model [2]	*91%*	97.8%	66.7%	92.7%	87.5%
Ant colony algorithm [73]	*84.3%*	93.9%	46.7%	87.5%	65.6%
Pixel-based Segmentation [1]	*84.8%*	93.4%	47.6%	88.5%	62.5%
Artificial Neural Network [74]	*83.4%*	93.8%	44.7%	86.5%	65.6%
Morphological Watershed [75]	*85.2%*	94.4%	48.9%	88%	68.8%
Presented FCM	*80.3%*	93%	38.5%	83.3%	62.5%
Presented FCM+GA	*95%*	97.9%	80%	96.4%	85.4%

**Table 2:** Quantitative comparative outcomes for segmentation of the retinopathy. This evaluation is conducted between our model and baseline studies. The assessments are based on Relative volume difference (RVD), Volume overlap error (VOE), and Dice similarity (DICE).

Method	Dice	RVD (%)	VOE (%)
Deep membrane [4]	*92%*	-2.46	6.16
Improved U-NET [6]	90*%*	3.74	5.78
PCNN model [2]	92*%*	3.55	6.41
Ant colony algorithm [73]	88*%*	-5.64	7.94
Pixel-based Segmentation [1]	88*%*	-4.91	7.42
Artificial Neural Network [74]	89*%*	5.37	8.12
Morphological Watershed [75]	87*%*	4.96	7.76
Presented FCM	86%	4.07	7.39
Presented FCM+GA	94%	2.32	4.28

## Data Availability

The dataset is available online: http://www.med.harvard.edu/AANLIB/.
